# Amphoteric composite of ZrP and N-doped porous carbon: Synthesis, characterization, and potential use for cycloaddition of CO_2_

**DOI:** 10.1016/j.heliyon.2023.e21353

**Published:** 2023-10-20

**Authors:** Yumiao Zhou, Dong Liang, Yuehua Yao, Lin Chen, Hongjiao Zhang, Yue Wu, Ting Zhao, Na Zhu

**Affiliations:** a School of Chemistry and Chemical Engineering, Shanxi Key Laboratory of High Performance Battery Materials and Devices, North University of China, Taiyuan 030051, PR China; b Shanxi Xinhua Chemical Defense Equipment Research Institute Co., Ltd., Taiyuan, 030008, PR China; c College of Environmental and Resource, Research Center of Environment and Health, Shanxi University, Taiyuan, 030006, PR China

**Keywords:** Zirconium phosphate, Carbon, Porous, CO_2_, Amphoteric, Tandem

## Abstract

Composites of amorphous ZrP and N-doped carbon were prepared in a one-step pyrolysis process instead of general post-loading technique. Owing to their mesoporous structure (6–10 nm) and Zr content (up to 41 wt%), the amphoteric materials have potential use in the cycloaddition of CO_2_ to epoxides, which is an acid‒base tandem process including the ring opening of epoxides and the addition of CO_2_. Substantial work has been done on how starting materials impact the structure and performance of composite materials. The coordination between metal and melamine has been confirmed, and it can be implanted in the melamine-polymer initiation of formation of porous metal-carbon materials. The composite catalysts exhibit amphoteric properties, present broad-spectrum adsorption, and finally produce carbonates via cycloaddition of CO_2_ to epoxides. It is remarkable that the multiple characteristics of porous solids are stabilized, and no significant loss of catalytic performance is observed after four cycles.

## 1 Introduction

Many functional materials in the recent past were deployed for CO_2_ capture, utilization, and storage (CCUS), and they witnessed the vital technologies to achieve carbon neutrality and improve climate quality. Porous materials, mainly reported as metal organic frameworks (MOFs), porous organic polymers (POPs), zeolites and carbons, have contributed to significant advancements in the design and implementation of gas molecule capture and subsequent conversion [1]. Carbonaceous materials with nanoporous structures are competitive contenders of other materials due to their feasibility of commercialization and functionalization. Porous carbons have the right characteristics for capturing CO_2,_ although the carbonaceous skeleton is believed stubborn and inert. The introduction of hybrid atoms and metal centers may alter the electronic configuration and surface chemistry of carbons, which can provide a perfect platform for the conversion of captured CO_2_ into value-added products. For example, N atoms are often doped into carbon-rich supports, not only strongly anchoring the individual metal centers but also modifying the electronic properties and enhancing the adsorption of greenhouse gases [2]. Relative to metals deposited on undoped supports, single-atom metals or nanocluster/nanoparticles on N-doped carbons could accomplish a uniform distribution and exhibit better performance in a variety of catalytic processes, including hydrogenation, dehydrogenation, oxidation, and coupling [3].

It is inspiring but challenged to achieve the metal-supported carbonaceous composites, especially the heteroatoms and metal nanoparticles simultaneously doped into the porous carbon framework. The general approaches for postloading metals on carbon supports include convenient deposition, precipitation, ion-exchange, and impregnation techniques [4]. Nonetheless, there is an uncontrollable aggregation of metals in pore channels. The electronic repulsion between metal precursors and the carbon surface also results in the failed loading of metals with uniformity. The porosity reduction and pore blockage occur with a high loading content, leading to a decreased efficiency of the supported catalyst. As another alternative, incipient-planting metals on carbon support have received growing attention due to their potential to achieve uniform distribution and higher metal density [5,6]. Notably, composite materials from the pyrolysis of MOFs have gained importance in electrochemistry and chromatography [7,8]. Different metals, after being imported into the porous organic precursors, are subjected to pyrolysis in nitrogen or argon. The metals chelating with organic ligands are converted to their corresponding metal oxides, and the whole metal-carbon composite system obtains multiple physicochemical characteristics at nanoscale proximity [9].

Carbon dioxide is chemically inert, and only active substances, such as hydrogen, alcohol, or epoxide, can make catalytic reactions proceed smoothly. The conversion of captured CO_2_ with epoxides was formerly considered an environment-friendly approach that could pave the way for the generation of carbonates, that is, an acid‒base tandem process involved opening the ring of epoxides and adding CO_2_ [10,11]. A porous catalyst with amphoteric character could be needed and designed by metal loading and heteroatom doping on the carbon matrix. ZrO_2_ provides advantages in possessing both acidic and basic characteristics for the adsorption of CO_2_ on surface hydroxyl groups [12,13]. Phosphorus zirconia (ZrP) has the active sites for remediation of pollutants, sensors of ammonia [14] and catalysts for alkylation [15], condensation [16], and dehydration [17]. The target of this research is to obtain high-loading and well-distributed ZrP on N-doped porous carbon via a facial one-step carbonization process. The amphoteric adsorbents or catalysts reveal multiple characteristics for tandem reactions, in which the strong metal-support interaction is crucial for determining the metal dispersion and porosity formation.

## 2 Experimental sections

### 2.1 Materials

Melamine (C_3_H_6_N_6_, 99 %), acetonitrile (CH_3_CN, >99 %), formaldehyde (CH_2_O, 35 %), phosphoric acid (H_3_PO_4_, ≥85 %), styrene oxide (C_8_H_8_O, 98 %), hydrogen peroxide (H_2_O_2_, 30 %), ammonium ceric (IV) nitrate (Ce (NH_4_)_2_(NO_3_)_6_, 99.0 %), and zirconium (IV) oxychloride octahydrate (ZrOCl_2_·8H_2_O, 99 %) were purchased from Shanghai Aladdin Bio-Chem Co., Ltd. All chemicals were used without further purification. CO_2_ and NH_3_ (>99 %) were supplied by Taiyuan Taineng Gas Co., Ltd.

### 2.2 Preparation of ZrP@N-doped carbon composites

The functionalized composites were prepared as follows. First, 2.5 g melamine was dissolved in 200 mL H_2_O at 80 °C. Different concentrations of zirconium solutions (specifically, 0.0, 0.8, 1.6 and 2.4 g ZrOCl_2_·8H_2_O in 20 mL H_2_O) were added dropwise into the above solution until white precipitates appeared. 1.6 mL of formaldehyde solution (37 wt%) and 3 mL of 85 % H_3_PO_4_ were sequentially supplemented. Then, the mixture was stirred for 6 h, and the solid products were retrieved after freeze-drying under a vacuum. The porous structures of the composites were preserved and carbonized at 450 °C for 3 h with nitrogen protection. The final products were called ZrP@N-doped carbon composites (**ZrPN/C-0.0/0.8/1.6/2.4)**.

### 2.3 Characterization of ZrP@N-doped carbon composites

Fourier transform infrared (FT-IR) spectra were recorded by a PerkinElmer Spectrum One FT-IR spectrometer in the range of 400–4000 cm^−1^. The specific surface areas were measured by N_2_ adsorption on Quantachrome Autosorb IQ. The microscopy images were collected by a Hitachi SU3500 scanning electron microscope (SEM) after deposition of a 3 nm-thick layer of Pd–Au by sputtering. The distribution of elements was observed by transmission electron microscopy (FEI TALOS F200X G2, Thermo Fisher). For the structural analysis of samples, X-ray powder diffraction (XRD) patterns were acquired using a Rigaku D/max-2550 X-ray diffractometer with Cu-Kα radiation (λ = 1.542 Å) at a potential of 40 kV. X-ray photoelectron spectroscopy (XPS) analysis was performed with a K-Alpha spectrometer (Thermo Fisher) equipped with a Cu Kα X-ray source. The zirconium content was determined on an Agilent 5110 (ICP‒OES) after the samples were digested in a mixture of 65 % HNO_3_ and 1 % HF in a microwave digester. Thermogravimetric curves (TG) were obtained with a Hitachi STA-200 analyzer in nitrogen protection at a heating rate of 10 °C·min^−1^. The temperature programmed desorption of CO_2_ (CO_2_-TPD) and NH_3_ (NH_3_-TPD) was conducted on an XQ 5080B analyzer (Tianjin). In brief, the sample was dried at 150 °C, cooled to 50 °C in helium and then saturated with 10 vol% CO_2_/He (or NH_3_/He) at 50 mL min^−1^. The gas flow switched back to helium and purged out the physiosorbed CO_2_ (NH_3_) on the surface of the catalyst. Finally, the sample was heated to 550 °C at a rate of 10 °C·min^−1^, and the signals of desorbed CO_2_ (NH_3_) were monitored by a TCD detector.

### 2.4 Catalytic ability of ZrP@N-doped carbon composites

The functionalized composites were subjected to CO_2_ fixation into styrene oxide. The batch reactions were carried out in a Parr reactor (supplied by Beijing Senlong SLM-100). Styrene oxide (1 mmol), acetonitrile (20 mL) and composite material (20 mg) were added into an autoclave reactor, which was evacuated, purged with CO_2_ and then placed under a constant pressure at 2 MPa for 15 min to equilibrate. The reactions were carried out at a specific temperature, and finally, the catalyst was separated after centrifugation. A small aliquot of the supernatant was taken for analysis by GC (supplied by Shanghai Haixin GC-950). Conversion of styrene oxide and selectivity of products were calculated based on the peak area normalization method.

## 3 Results and discussion

### 3.1 Characterization of ZrP@N-doped carbon composites

**Fig. 1:**
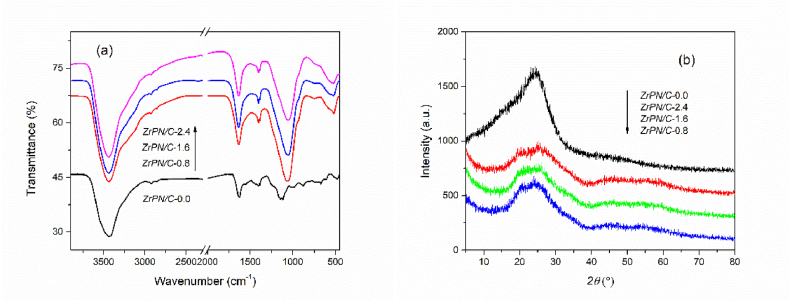
(a) FTIR spectra and (b) XRD patterns of ZrP@N-doped carbon composites.

Successful ZrP implantation on the N-doped carbons can be confirmed by the FTIR and XRD spectra. As shown in Fig. 1a, a broad band at approximately 3430 cm^−1^ is generated by the stretching vibration of N–H groups. Additionally, the peak at 1400 cm^−1^ can be recognized as the bending vibration of N–H groups. The common peak at 1635 cm^−1^ in all samples represents aromatic C=C stretching in the carbon framework. There are two typical bands at 1035 cm^−1^ and 930 cm^−1^, derived from the deformation and asymmetric stretching vibrations of O–P–O fragments. In addition, the small and weak band at 750 cm^−1^ could be ascribed to the symmetric stretching vibrations of P–O bonds [18]. The Zr–O stretching peak is clear at 560 cm^−1^ [19]. However, these bands are not seen in ZrPN/C-free samples (ZrPN/C-0.0). Meanwhile in Fig. 1b, the differences in characteristic X-ray diffraction peaks are not significant because of the uniform distribution of metal NPs on the functionalized composites. Broad characteristic peaks at 25.4° and 44.1° are found in the XRD curves of all samples and attributed to the (002) and (100) crystal planes of carbon materials [20,21], respectively. The typical peak is observed at 19.7° and slightly increased with the content of ZrP, which could be due to the diffraction of the (110) plane of ZrP (JCPDS 33–1482) [22]. Compared with pristine carbon, the amorphous nature of ZrPN/C exhibits some broad and low-intensity peaks at 20–60°, which slightly increase with the content of ZrP [23,24], but it does not seem to be easily recognized in this work.

**Fig. 2:**
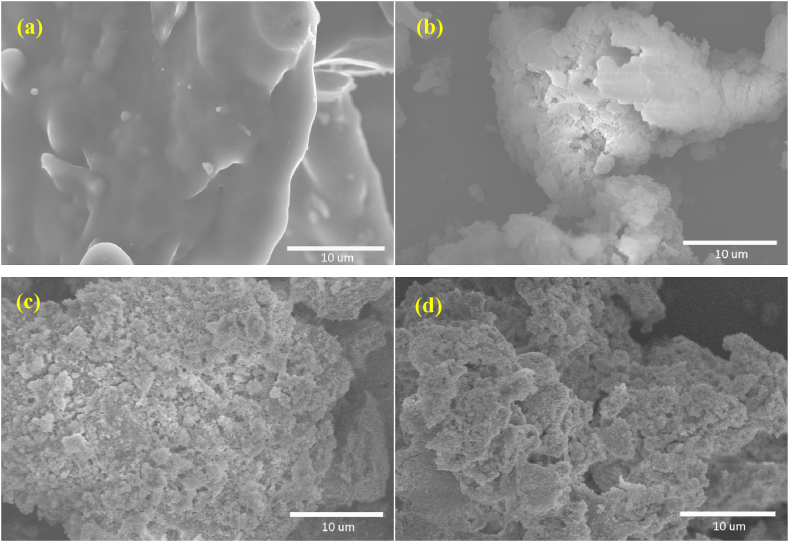
SEM images of ZrPN/C-0.0, 0.8, 1.6, and 2.4 (a, b, c, d).

**Fig. 3:**
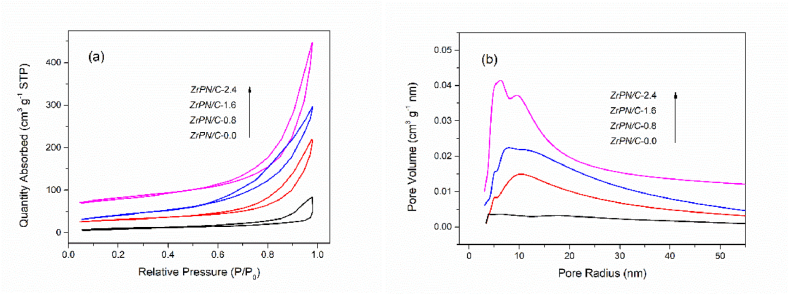
(a) N_2_-isothermal adsorption curves and (b) pore-radius distributions of ZrP@N*-doped* carbon composites.

The morphological characteristics of functional composites with increasing ZrP loading were observed by SEM images (in Fig. 2), all showing porous structures except for the metal-free samples. The metal-free precursors were produced via the condensation process between melamine and formaldehyde. As displayed in Fig. 2a, nonporous carbons with a low surface area were obtained after carbonization. However, the incorporation of ZrP significantly changed the porosity of carbon materials (in Fig. 2b–d). Moreover, the N_2_-isothermal adsorption curves and pore radius distributions are described in Fig. 3 (a, b). Their specific surface areas and mean pore radius using BET and BJH methods were calculated and are listed in Table 1. The porosity of ZrP-loaded composites is more prominent than that of metal-free carbons. Larger surface areas and larger pore radius were obtained with more metallic content, whereas the overloaded samples also may undergo the pore blockage and configurational destruction [25]. These cavities in ZrPN/C-1.6 and ZrPN/C-2.4 were controlled at the mesoscale of 6∼8 nm, which provided easily accessible channels for CO_2_ and epoxides.

**Table 1:** The structural and amphoteric properties of ZrP@N-doped carbon composites.

Samples	Surface Area (m^2^ g^−1^)	Pore Radius (nm)	Total Volume (cm^3^ g^−1^)	Zr- Content (wt.%)	Q_*CO2*_ (mmol g^−1^)	Q_*NH3*_ (mmol g^−1^)
ZrPN/C*-*0.0	49	3.8	0.15	0.0	6.7	0.6
ZrPN/C*-*0.8	76	9.7	0.32	19.5	4.2	3.1
ZrPN/C*-*1.6	152	7.8	0.64	41.3	5.0	4.7
ZrPN/C-2.4	150	6.6	0.55	53.4	4.7	4.0

Melamine is a heterocyclic triazine that has been criticized in clinical medicine for the induction of kidney stones, in which melamine can be easily chelated with soluble metal ions. Melamine is a nitrogen-rich ligand that has been widely used for the coordination of metal-organic frameworks (such as Pd, Cu, and Ag) [[26], [27], [28]]. The complex of melamine with zirconium (Ⅳ) ions is shown and characterized in the supplementary information (Fig. S1). This result indicated the presence of strong metallic-organic interactions between the zirconium ions and the N-containing polymer resins, which probably immediately played a large role in the formation of nanoporous ZrP@N-carbon composites. The combination between metallic centers and organic ligands are well preserved and retained under the pyrolysis process. The local organics crosslinked with metallic centers decompose faster than metal-free organics and etch out a quantity of nanocavities in the carbonaceous framework. This combination could protect them against collapse during carbonization [29]. Thus, the coordinating matrix of the metal-melamine polymer served as a precursor for well-dispersed ZrP on N-containing carbons, showing advantages in automatic and one-step doping of heteroatoms and metals into the carbonaceous framework compared with previous postloading techniques.

### 3.2 Amphoteric properties of ZrP@N-doped carbon composites

**Fig. 4:**
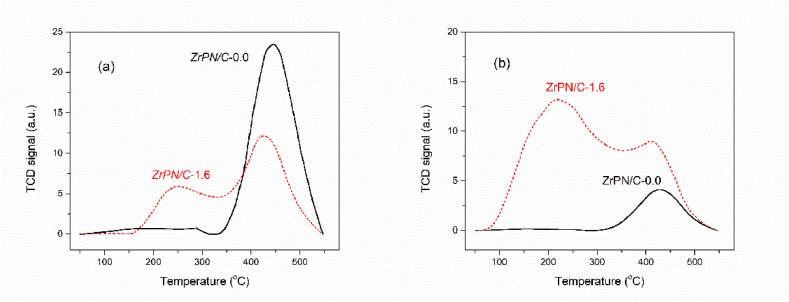
(a) CO_2_-TPD and (b) NH_3_-TPD curves of ZrPN/C*-*1.6 and ZrPN/C*-*0.0.

The amphoteric character of the composite catalysts could be examined with broad-spectrum adsorption on CO_2_ and NH_3_ in Fig. 4 (a, b). The CO_2_-TPD and NH_3_-TPD results suggest that the composite of ZrP and N-doped carbon retains the coexistence of acidic and basic sites, which facilitate the tandem reaction between epoxides and CO_2_. The desorption peaks of the NH_3_-TPD profile located at 250 °C and 425 °C can be assigned to moderate and strong acid sites, respectively. The desorption peaks of the CO_2_-TPD profile at 220 °C and 410 °C indicate moderate and strong basic strengths [30,31]. The catalytic activity was primarily related to the copresence of acid‒base sites located on the composite catalyst, and the synergistic mechanism between them determines the yield of the cyclo-carbonates. As confirmed by the analysis of metal-free ZrPN/C*-*0.0, the poorest acidic property (NH_3_ as probe) is not favored for the opening of epoxides, although the strongest basic capacity (CO_2_ as probe) was observed, which could explain the minimal conversion of CO_2_.

The quantity of adsorption is listed in Table 1. Specifically, the metal-free samples (ZrPN/C-0.0) showed a high capacity for CO_2_ (6.7 mmol g^−1^) and a poor capacity for NH_3_ (0.6 mmol g^−1^). The introduction of ZrP on the carbon framework significantly increased the adsorption ability for basic gases. The adsorption capacity of ZrPN/C adsorbents for NH_3_ increased by 6–8 times, and the capacity for CO_2_ remained high compared to the metal-free samples. ZrPN/C-1.6 showed the best performance with an amphoteric capacity of 5.0 mmol g^−1^ for CO_2_ and 4.7 mmol g^−1^ for NH_3_. The results suggested that the opposite active sites could coexist in the same composite and enable the carbon materials to have a broad-spectrum adsorption ability for CO_2_ and NH_3_.

**Fig. 5:**
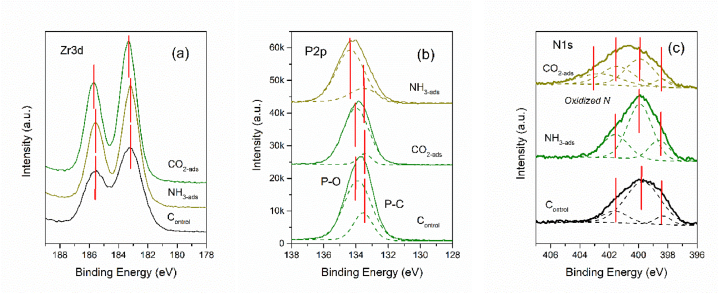
XPS spectra of Zr3d (a), P2p (b) and N1s (c) (including control and samples after adsorption of CO_2_ and NH_3_).

The XPS spectra are shown in Fig. 5. The adsorption of ammonium and carbon dioxide on the surface brought out distinct changes in the XPS spectra of Zr3d, P2p and N1s. Compared with the control and NH_3_-ads samples (after adsorption of NH_3_), we found that zirconium could be involved in the adsorption of CO_2_. The Zr–O bond in the CO_2_-ads sample (after adsorption of CO_2_) may react with CO_2_ to produce zirconium carbonate, which causes the core binding energies of Zr3d_5/2_ (183.2 eV) and Zr3d_3/2_ (185.6 eV) to shift up at 0.15 eV [32]. The P2p signal fitting curves can be deconvoluted into two regions at 133.5 and 134.0 eV due to P–C and P–O bonds, respectively [33,34]. The weak presence of P–C peaks suggests that the phosphorus atoms were partially incorporated into the carbon framework. The dominant P–O bonds were significantly affected at approximately 0.3 eV, but was offset by the adsorption of NH_3_, but no obvious changes were found in the CO_2_-adsorbed samples and control samples. In the control and NH_3_-ads samples, the high-res spectra of N 1 s were deconvoluted into three peaks, which corresponded to graphitic N (401.7 eV), pyrrolic N (399.8 eV) and pyridinic N (398.4 eV) [35]. CO_2_ adsorption caused a new peak at 402.6 eV, which possibly arose from oxidized pyridinic nitrogen [36,37]. A relatively high portion of pyrrolic groups were consumed off, suggesting that the C–N bonds might provide more active sites for CO_2_ adsorption over pyridinic and quaternary N atoms.

### 3.3 Porosity formation in the one-step pyrolysis process

**Fig. 6:**
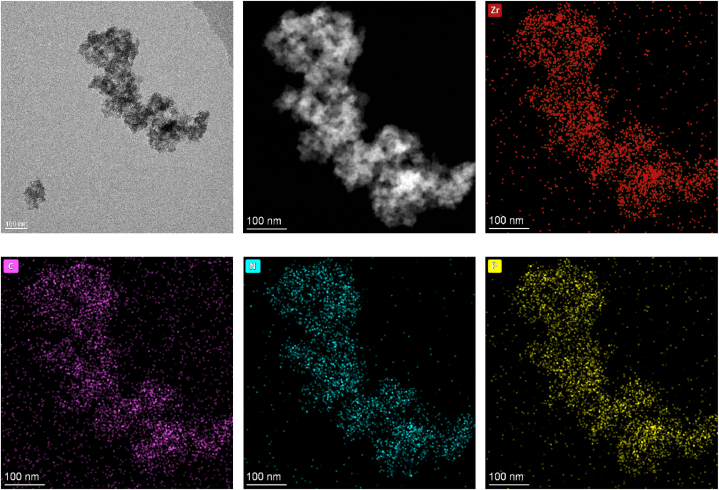
(a) TEM image, (b) dark field and (c–f) element map of ZrPN/C*-*1.6.

It has been noted that unsupported adsorbents are not as active as supported adsorbents. This is due to the presence of active atoms on the carbon frameworks. ZrPN/C-1.6 was chosen for further characterization because it had the best performance in the capture of gas molecules. The uniform doping of N, P and Zr atoms in the carbon materials was confirmed through TEM analysis in Fig. 6(a–f). The heteroatoms generally act as electron acceptors or donors when anchoring the metals, and the refined interaction between them significantly assures the good distribution of metals on the support surface. In addition, the loading content of zirconium was as high as 41 wt% by ICP‒OES, which is one of the highest values among the known supported carbons (Table S1).

**Fig. 7:**
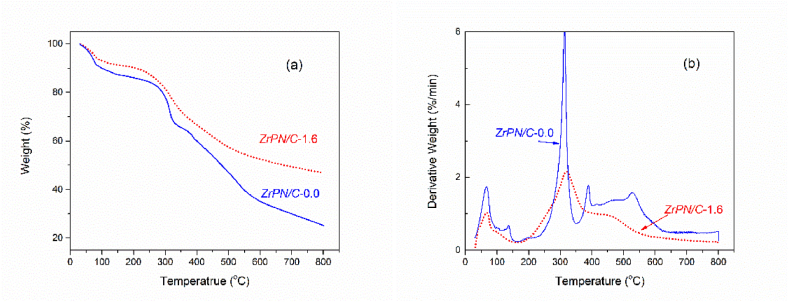
TG-DTG analysis for precursors of ZrPN/C-1.6 and metal-free ZrPN/C-0.0.

Controlling the pyrolysis conditions to avoid coordination destruction and metal-NP aggregation remains a challenge. For the synthesis of well-dispersed functional composites, metal and precursors are dissolved and uniformly implanted together into the continuous frameworks. Thermogravimetry (TG) analysis of the composite precursors was conducted in Fig. 7a, suggesting that the pyrolysis temperature was preferred in the range of 450–500 °C. In the pyrolysis process at temperatures higher than 550 °C, the breaking of coordination bonds and the decomposition of triazine might be accompanied by the sintering and agglomerating of metal nanoparticles on the pore channels. A distinct weight loss occurred at approximately 320 °C, which was caused by the release of small molecules such as coordinated formaldehyde and evaporated melamine within structural defects [38]. That process results in the formation of the nanoscale cavity and then is enlarged when heating to 450 °C. On the other hand, there are multiple decomposition peaks on the metal-free N-doped polymers (Fig. 7b). The decomposition peak at 320 °C in ZrPN/C-1.6 was taken in advance at 312 °C because of the better thermoconductivity of carbon materials. With increasing temperature, there was a broad plateau between 389 °C and 528 °C. However, this plateau moves to a narrow range of 400 °C and 450 °C after incorporating the metal centers, which indicates the regularity of porous composites. Herein, the key issue is the thermal stability of the interaction or chelation between metal ions and supports in the polymer structure [39]. The formation of rigid and porous composite polymers before carbonization (as seen in Fig. S2) shows great benefits in confining the metal NPs with controlled sizes and replicating the porous frameworks.

### 3.4 Catalytic ability of ZrP@N-doped carbon composites

The effect of catalytic conditions on CO_2_ conversion was conducted using ZrPN/C*-*1.6 as the catalyst because of its substantial capture of gas molecules. The cyclic carbonate was the main product, and only a small quantity of byproducts was occupied with styrene glycol from hydrolysis. In detail, the pyrolysis temperature, the reaction temperature and pressure, the reaction time, and the usage of styrene oxide are all shown in the Supplementary Information (Fig. S3). Finally, the optimal pyrolysis temperature was found at 450 °C, and the suggested reaction conditions were at 150 °C for 5 h with 1.5 MPa CO_2_ and 100 μL styrene oxide in 20 mL acetonitrile.

**Fig. 8:**
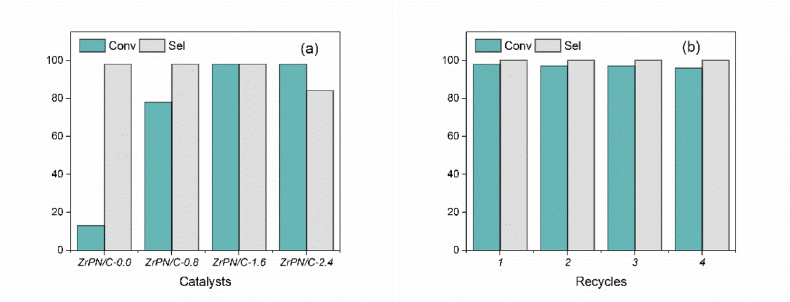
Results of catalytic and recycling experiments (20 mg catalysts, 20 mL acetonitrile, 1.5 MPa CO_2_ and 100 μL styrene oxide were mixed and reacted for 5 h).

As shown in Fig. 8a, the conversion of styrene oxide is found to increase from 16 % to 98 % with the increase in ZrOCl_2_·8H_2_O usage from 0 g to 2.4 g. This can probably be attributed to (i) the enhancement in BET surface area, (ii) the increase in ZrP active sites, and (iii) the increase in CO_2_ capture ability. Meanwhile, the carbonate selectivity decreases from 98 % to 81 % with increasing ZrOCl_2_ usage from 1.6 g to 2.4 g. As a result, the carbonate yield follows a volcanic shape with increasing Zr loading, reaching the highest value at 98 % for ZrPN/C-1.6.

### 3.5 Reusability and stability of ZrP@N-doped carbon composites

**Fig. 9:**
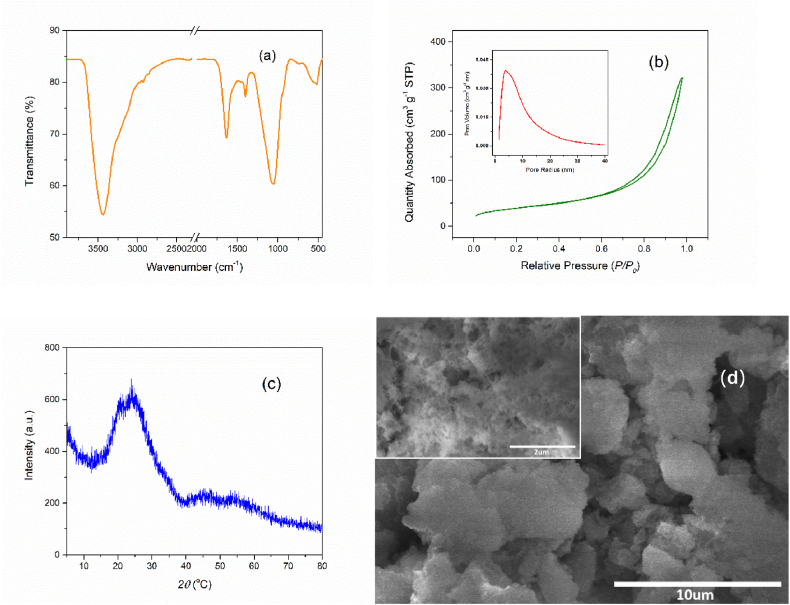
Characterization of the recycled catalyst.

The reusability and stability of the composite catalyst were also investigated and are shown in Fig. 8b. It can be suggested that the ZrP-loaded porous carbon skeletons are stabilized and that the catalytic performance is maintained at a steady level even after recycling four times. Upon completion of each cycle, the catalyst was separated from the solution by filtration, washed twice with 5 mL of absolute alcohol, and then involved in the next cycle. Investigation of the chemical composition, crystalline structure, porous skeleton, and microscopic properties of the reused catalysts (after 4 cycles) was carried out and is presented in Fig. 9(a–d). The characteristic peaks at 1035 cm^−1^ and 930 cm^−1^ were observed owing to the O–P–O fragments. The typical Zr–O stretching band was clear at 560 cm^−1^. The mesopore distribution was retained, as proven by the N_2_ physisorption and BJH desorption results. There were no characteristic peaks shown on XRD, and the metal particles dispersed in the carbon skeleton did not undergo significant aggregation. It is clearly seen in the SEM images that the porous morphology of the reused samples is almost the same as that of the fresh samples. The investigations on the recycled catalysts indicated the stable interaction between the metals and the N-doped carbons, and furthermore, the elemental content of zirconium was determined to be 39.8 %, showing a small loss compared to the fresh catalyst.

### 3.6 Plausible tandem mechanism of the cycloaddition of CO_2_

**Scheme 1:**
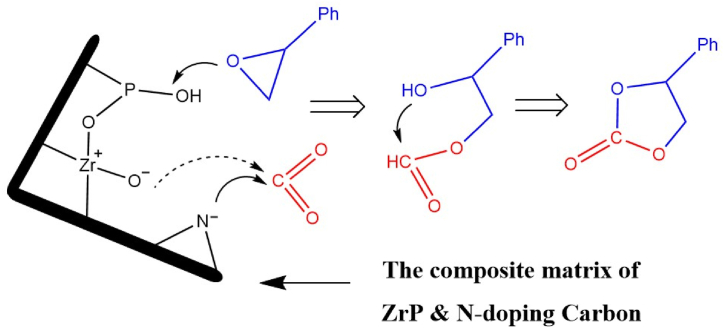
Proposed tandem mechanism of the composite catalysts in the cycloaddition of CO_2_.

A plausible mechanism was proposed above and is described in Scheme 1. The synergistic effect of amphoteric active sites is important for the cycloaddition reaction between epoxides and CO_2_. The phosphoric element adjacent to the zirconium center provides a strong acidic center, which ensures the instant opening of ring epoxides. The nitrogen element in the carbon skeleton provides abundant basic centers for capturing and activating CO_2_, and zirconium itself can also provide certain basic sites [40]. The intermediates of the opened epoxide were vulnerable to the captured carbon oxides, and finally, the cyclo-carbonates were formed. Further contrast experiments were conducted on amorphous zirconium phosphate and N-doped carbons (pyrolyzed with melamine resin), in which CO_2_ did not significantly convert into the valuable chemicals (in Fig. S4).

## 4 Conclusion

In this presentation, there is established a coordination between melamine and zirconium ions. The strong coordination imported to the melamine polymers and protected the well-distribution of metal NPs. Through a facile one-step carbonization process, the high loading NPs can be uniformly dispersed into the porous N-doped carbon. The amphoteric composite has the mesoporous structure and high Zr-content, which offers exciting potential for the tandem reaction of CO_2_ under halogen-free conditions. In a proposed mechanism, the amorphous ZrP and N-doped carbon in the composite should function synergistically. The fixed coexistence of acid-base sites endows the porous composites with the distinctive reusablity and catalytic ability, that is stemmed from an innovative and automatic implantation of metal NPs and heteroatoms.

## Data availability

The data that support the findings of this study are available from the corresponding author, [Dr. Liang], upon reasonable request.

## Fundings

This study was supported by 10.13039/501100001809National Natural Science Foundation of China (No. 22176117) and 10.13039/501100004480Natural Science Foundation of Shanxi Province (No. 201901D111171).

## CRediT authorship contribution statement

**Yumiao Zhou:** Data curation, Methodology, Writing – original draft. **Dong Liang:** Conceptualization, Project administration, Writing – review & editing. **Yuehua Yao:** Formal analysis, Software, Visualization. **Lin Chen:** Investigation, Resources. **Hongjiao Zhang:** Software. **Yue Wu:** Resources. **Ting Zhao:** Formal analysis. **Na Zhu:** Supervision, Validation.

## Declaration of competing interest

The authors declare that they have no known competing financial interests or personal relationships that could have appeared to influence the work reported in this paper.
