# Supplementary material for: Amphoteric composite of ZrP and N-doped porous carbon: Synthesis, characterization, and potential use for cycloaddition of CO2
Source: Heliyon. 2023 Oct 20;9(11):e21353. doi: 10.1016/j.heliyon.2023.e21353 (PMC10623289; doi:10.1016/j.heliyon.2023.e21353)
Supplement: Multimedia component 1 [file mmc1.docx]

Supplementary Data

Amphoteric composite of ZrP and N-doped porous carbon: Synthesis, characterization, and potential use for cycloaddition of CO_2_

Yumiao Zhou^1^, Dong Liang^1^, Yuehua Yao^1^, Lin Chen^1^, Hongjiao Zhang^1^, Yue Wu^2^, Ting Zhao^2^, Na Zhu^3^

*1.* *School of Chemistry and Chemical Engineering, Shanxi Key Laboratory of High Performance Battery Materials and Devices, North University of China, Taiyuan 030051, P. R. China;*

*2. Shanxi Xinhua Chemical Defense Equipment Research Institute Co., Ltd., Taiyuan, 030008, P. R. China*

*3. College of Environmental and Resource, Research Center of Environment and Health, Shanxi University, Taiyuan, 030006, P. R. China*

* The first two authors took equal contributions for this article.

Dr. Liang is the corresponding author and his email is [liangdong@nuc.edu.cn](mailto:liangdong@nuc.edu.cn).

***1. The complex of Melamine to zirconium by hydrothermal process***

Fig. S1 FT-IR and XRD result of Zr-melamine complex

**Preparation**

Melamine (2.5 g, 0.02 mol) was dissolved in 200 mL deionized water at 80 ℃, and then ZrOCl_2_ 8H_2_O (1.6 g, 0.005 mol) in 20 mL H_2_O was dropwise added. After 3-5 h reaction, the suspended white solid was collected by filtration, washed by distilled water, and dried at 60 ℃ for 24 h under vacuum. The element analysis of products is C 0.286, N 0.667, and H 0.047. The content of Zr was determined to 15 wt.% by ICP-OES. Finally, the molar ratio of melamine to zirconium is calculated close to 4/1.

**Characterization**

Selected IR data (cm^-1^): 3465, 3344, 3223, 2905, 1647, 1540, 1368, 685. The set of peaks that are related to amine groups are N-H stretching (3000-3500 cm^-1^), asymmetric N-H stretching (3465 and 3344 cm^-1^), symmetric N-H stretching (3223 cm^-1^) and N-H deformation (1647 cm^-1^). The peaks at 2905 cm^-1^ and 2837 cm^-1^ are associated with the stretching of C-H. Characteristic peaks of triazine are 1540, 1368 and 685 cm^-1^. The adsorption at 503 cm^-1^ could be related to the stretching of Zr-O. Interestedly, X-ray diffraction peaks of Zr-melamine complex are mostly attributed to the characteristic peaks of melamine. Hu et al [1] suggested these typical peaks would disappear after synthezing the nitrogen-doped carbon from meleamine under the carbonation. There is a broad peak around 50° that might be caused by Zr incorporation.

[1] Chuangang Hu, Ying Xiao, Yang Zhao, Nan Chen, Zhipan Zhang, Minhua Cao, Liangti Qu. Highly nitrogen-doped carbon capsules: scalable preparation and high-performance applications in fuel cells and lithium ion batteries[J]. ***Nanoscale* 5** (2013) 2726-2733. https://doi.org/10.1039/C3NR34002C

***2. The porous polymer of Melamine resin to zirconium before pyrolysis***

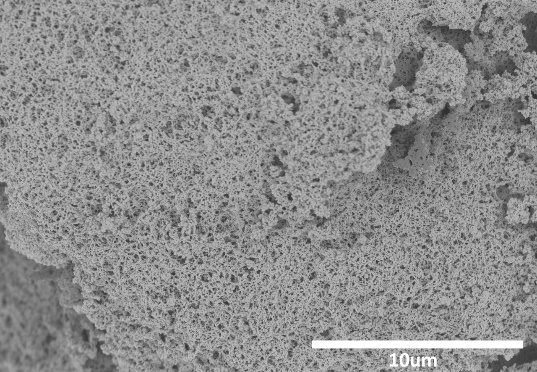

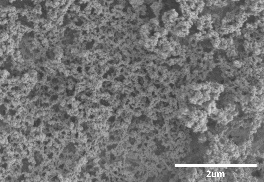


Fig. S2 the porous polymer of melamine resin to zirconium before pyrolysis

***3. The optimum parameters for the CO_2_ conversion on the composite catalysts***

Fig. S3 Effect of the catalytic conditions (a, pyrolysis temperature; b, reaction temperature; c, reaction pressure; d, usage of styrene oxide) using 20 mg catalyst and 20 mL acetonitrile for 5 h.

***4. Preparation and catalytic ability of amorphous ZrP***

Amorphous-ZrP (am-ZrP) was prepared via a room-temperature precipitation method reported by Junhua Xu’s works [1,2]. 10.6 g of ZrOCl_2_·8H_2_O was firstly dissolved in 100 mL of 2 M HCl and then 100 mL of 1.25 M H_3_PO_4_ was added. The resulting precipitated solid was kept over-night and filtered, washed with 1.25 M H_3_PO_4_ to clear off the unbound Cl^−^ ions. Finally, the am-ZrP product was rinsed with deionized water until the pH of the filtrate reached around 3, and dried in an oven at 60 °C for 24 h.

The above amorphous ZrP and N-doping carbon (ZrPN/C-0.0) were applied in the catalytic conversion of CO_2_ as following as the same procedures for ZrPN/C composites. The reaction conditions were built at 150 ℃ for 5 h with 1.5 MPa CO_2_, 20 mg catalyst and 100 μL styrene oxide in 20 mL acetonitrile. A small aliquot of the supernatant was taken to be analyzed by GC (supplied by Shanghai Haixin GC-950). Conversion of styrene oxide and selectivity of products were calculated based on the peak area normalization method. Finally, the results were described and compared in Fig. S4. of all it is noted that the selectivity of carbonate on the am-ZrP and N-carbon (ZrPN/C-0.0) are not satisfied compared with the composite catalyst. The acetophenone styrene glycol and benzoic acid are the main byproducts instead of the carbonate.

Fig. S4 the contrast experiments on single one and their composite

[1] Junhua Xu, Risto Koivula, Wenzhong Zhang, Elmo Wiikinkoski, Sami Hietala, Risto Harjula. Separation of cobalt, neodymium and dysprosium using amorphous zirconium phosphate[J]. Hydrometallurgy 175 (2018) 170-178. https://doi.org/10.1016/j.hydromet.2017.11.010

[2] Junhua Xu, Sami Virolainen, Wenzhong Zhang, Jukka Kuva, Tuomo Sainio, Risto Koivula. Polyacrylonitrile-encapsulated amorphous zirconium phosphate composite adsorbent for Co, Nd and Dy separations[J]. Chemical Engineering Journal 351 (2018) 832–840. https://doi.org/10.1016/j.cej.2018.06.112

***5. Well distribution of amorphous ZrP on the N-doped carbon materials***

Fig. S5 The HR-TEM images and EDX analysis of ZrPN/C-1.6


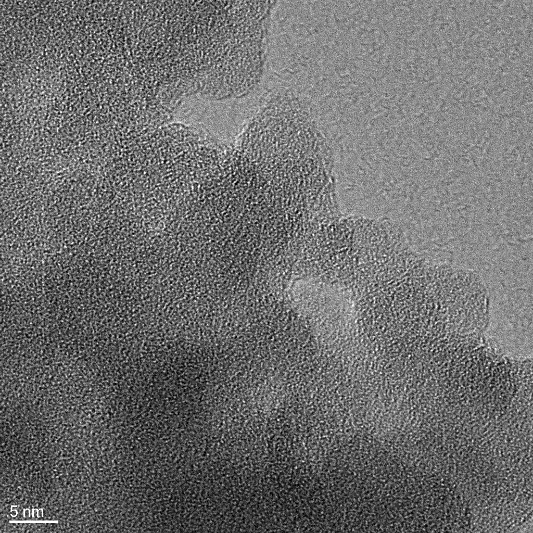

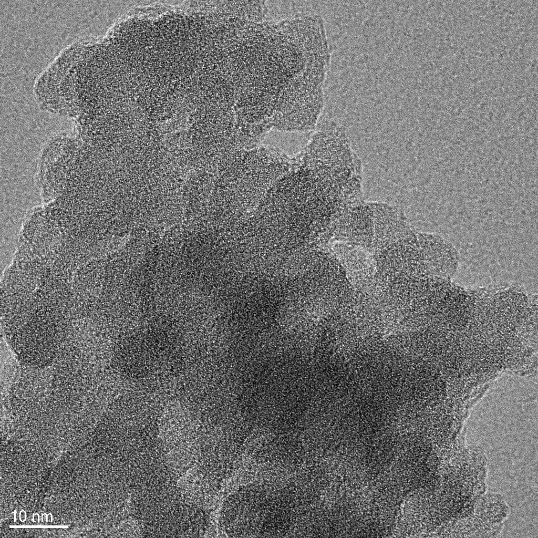

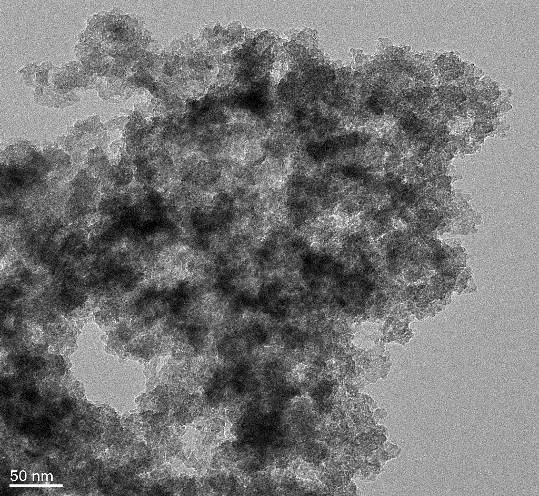

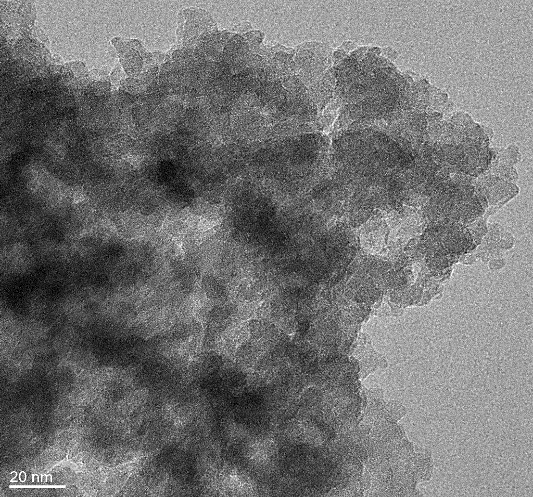

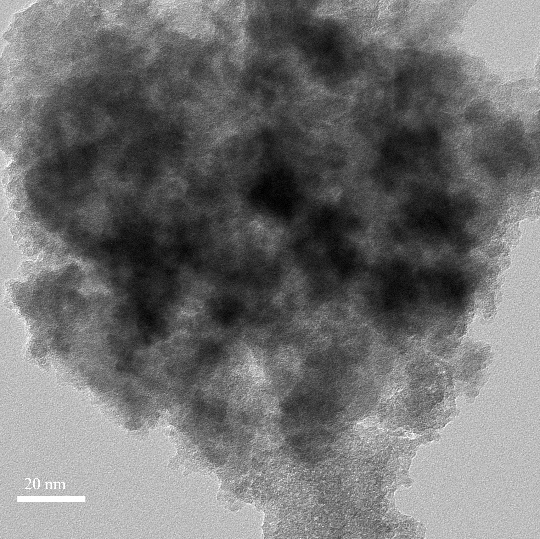

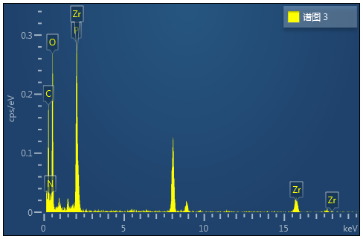

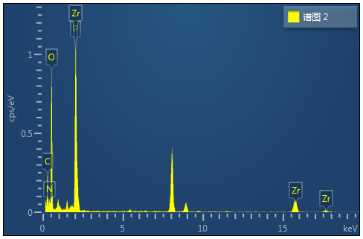


C: 11.26 wt.%

N: 1.59 wt.%

O: 40.76 wt.%

P: 22.37 wt.%

Zr:24.02 wt.%

C: 12.57 wt.%

N: 3.24 wt.%

O: 38.03 wt.%

P: 22.52 wt.%

Zr:23.64 wt.%

***6. Amphoteric gas capture via the volumetric breakthrough apparatus***


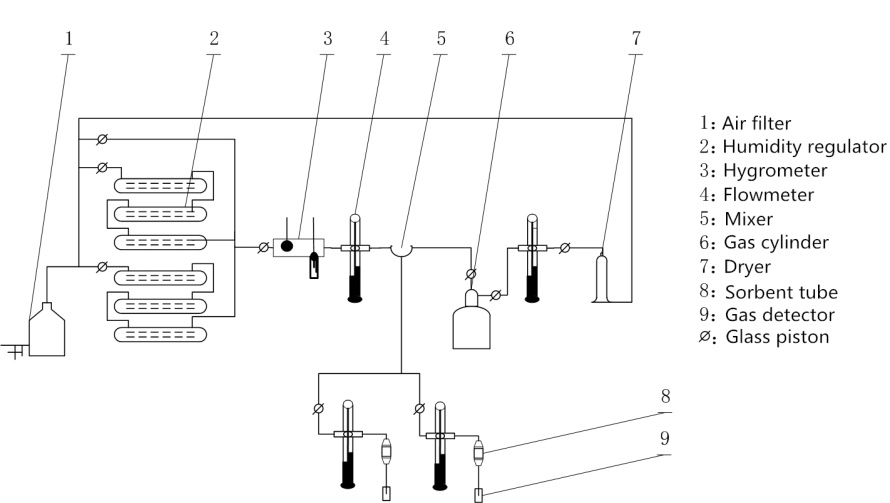


Fig. S6 Schematic diagram of volumetric breakthrough apparatus

Two typical gases were selected in this work, including carbon dioxide (acidic) and ammonia (basic). As it is shown Fig S6, the adsorption and breakthrough analysis were performed using a custom-built apparatus using a column of 10 cm height and 0.5 cm diameter. Filled with the sample amounts of 300 mg in a mixed 1:1 wt. ratio to silica beads in order to avoid large pressure drops through the column [18]. The gas detectors were installed at the pipeline outlet and recorded at intervals. The samples were previously activated at 60 ℃ under vacuum for 12 h before test. The column was filled with the solid particles in a clean glovebox, and subsequently purged with a helium flow (10 mL min^-1^) for at least 1 h once connected to the apparatus.

Adsorption capacities for the adsorbents were determined at a low concentration and in a balance of nitrogen (separately at 0.12 mmol mL^-1^ NH_3_ and 0.06 mmol mL^-1^ CO_2_). The adsorption capacity (mmol adsorbate/g adsorbent) was calculated by the following formula,

$Q=\frac{F(C_{t}-C_{o})t}{m}$

where *C_0_* is the feed concentration in units of mol m^-3^ and *C_t_* is the effluent concentration at time t. The mass of adsorbents (m) was stuffed into column and the volumetric flow rate of gas through the adsorbent bed (F) was held constant at 10 mL min^-1^. The concentration of out-flowing gas will return to the level of inlet after the gas penetration through the column.







Fig. S7 Breakthrough plots of the composite catalysts separately using a concentration of 0.06 mmol mL^-1^ CO_2_ (a) and 0.12 mmol mL^-1^ NH_3_ (b) in a balance of nitrogen and pre-saturating the bed with helium prior to flowing.

Fig. S7 shows the CO_2_ and NH_3_ breakthrough curves for the composite materials. As can be seen, the CO_2_ breakthrough time for metal-free sample is obviously higher than the breakthrough time for the metal-loading samples but its NH_3_ breakthrough time is significantly lower than the others, that is attributed to the insufficiency of acidic sites. Results show CO_2_ and NH_3_ begins to break through the packed column of ZrPN/C-1.6 separately around 32 and 27 mins, whereas the ZrPN/C-0.8 lasts over 21 and 16 mins before the toxic gas begins eluting. One explanation for this is the difference in specific surface area. The surface area of ZrPN/C-1.6 is noted two times larger than that of ZrPN/C-0.8.

Another potential limitation in the composite catalysts is the abundance and distribution of active sites stemmed from the metal-loading process. The adsorption efficiency for ZrPN/C-2.4 slightly deceases with more metal loading, owing to the possible metal-sintering and pore blockage. It can be supposed that the materials with high capacity and mass-transfer efficiency generally possess nanoporous structure and well-dispersive metals. The broad-spectrum adsorption for the composite materials is sufficient to warrant further tandem catalysis.

***7. Discussing zirconium content of the previous samples***

Table S1 Summary of Zr loading supported carbons before this article

|  | Method | Zr content | Support | Textural properties |
| --- | --- | --- | --- | --- |
| ZrO_2_/MWCNTs ^[1]^ | Hydrothermal synthesis in autoclave reactor | 42.79 wt.% | Multi-walled CNTs  (MWCNTs) | BET surface area of 164 m^2^⋅g^−1^  Pore volume of 0.70 cm^3^⋅g^-1^ |
| ZrOx/AC ^[2]^ | Impregnation method | 0.77 wt.% | Activated carbon (AC) | BET surface area of 867 m^2^⋅g^−1^  Pore volume of 0.332 cm^3^⋅g^-1^ |
| zirconium oxide supported activated carbon (ZrSAC5) ^[3]^ | Microwave combustion method | 51.37 wt.% | Activated carbon (AC) | BET surface area of 69.84 m^2^⋅g^−1^  Pore volume of 0.04 cm^3^⋅g^-1^ |
| Zr-loaded P-containing mesoporous activated carbon ^[4]^ | Impregnation method | 14.9 wt.% | Activated carbon (AC) | BET surface area of 1190 m^2^⋅g^−1^  Pore volume of 0.88 cm^3^⋅g^-1^ |
| Zr-loaded on C_3_N_4_ ^[5]^ | Impregnation method | 8.29 wt.% | Carbon nitride | BET surface area of 70.1 m^2^⋅g^−1^ |
|  |  |  |  |  |

Ref.

[1] Mu Shiyun, Liu Kai, Li Hong, Zhao Zhenyu, Lyu Xiaoqi, Jiao Yilai, Li Xingang, Gao Xin, Fan Xiaolei. Microwave-assisted synthesis of highly dispersed ZrO2 on CNTs as an efficient catalyst for producing 5-hydroxymethylfurfural (5-HMF)[J]. Fuel Processing Technology 233 (2022) 107292. https://doi.org/10.1016/j.fuproc.2022.107292

[2] Velazquez-Jimenez Litza Halla, Hurt Robert H., Matos Juan, Rangel-Mendez Jose Rene. Zirconium-carbon hybrid sorbent for removal of fluoride from water: oxalic acid mediated Zr(IV) assembly and adsorption mechanism[J]. Environmental Science and Technology 48 (2014) 1166-1174. https://doi.org/10.1021/es403929b

[3] Suresh P., Vijaya J. Judith, Kennedy L. John. Photocatalytic degradation of textile-dyeing wastewater by using a microwave combustion-synthesized zirconium oxide supported activated carbon[J]. Materials Science in Semiconductor Processing 27 (2014) 482-493. https://doi.org/10.1016/j.mssp.2014.06.050

[4] Palomo J., Rodríguez-Cano M.A., Rodríguez-Mirasol J., Cordero T. On the kinetics of methanol dehydration to dimethyl ether on Zr-loaded P-containing mesoporous activated carbon catalyst[J]. Chemical Engineering Journal 378 (2019) 122198. https://doi.org/10.1016/j.cej.2019.122198

[5] Lin Wenwen, Yao Siyu, Chen Hao, Li Shenglai, Xia Yang, Yao Yuan, Li Jing, Cheng Dangguo, Fu Jie. A new trick on an old support: Zr in situ defects-created carbon nitride for efficient electrochemical nitrogen fixation[J]. Journal of Energy Chemistry 53 (2021) 109-115. https://doi.org/10.1016/j.jechem.2020.05.013

***8. Elemental composition of samples via the XPS analysis***

Fig. S8 the XPS survey of ZrPN-C samples

Table S2 Summary of element composition with am-ZrP@N-doped carbon materials

|  | C (At. %) | N (At. %) | O (At. %) | P (At. %) | Zr (At. %) |
| --- | --- | --- | --- | --- | --- |
| ZrPN/C-0.0 | 72.44 | 19.03 | 8.53 | 0.00 | 0.00 |
| ZrPN/C-0.8 | 52.76 | 16.06 | 23.89 | 4.28 | 3.01 |
| ZrPN/C-1.6 | 29.34 | 7.68 | 44.26 | 10.09 | 8.22 |
| ZrPN/C-2.4 | 26.11 | 4.37 | 47.15 | 12.12 | 10.25 |

*At. % means Atomic %.
